# The effect of apatinib combined with chemotherapy or targeted therapy on non‐small cell lung cancer in vitro and vivo

**DOI:** 10.1111/1759-7714.13162

**Published:** 2019-09-04

**Authors:** Mingtao Liu, Xiuxiu Wang, Hui Li, Lisheng Xu, Lijun Jing, Peng Jiang, Baoyi Liu, Yu Li

**Affiliations:** ^1^ Department of Pulmonary Medicine, Qilu Hospital Shandong University, Jinan Shandong China; ^2^ Department of Pulmonary Medicine Binzhou People's Hospital, Binzhou Shandong China; ^3^ Department of Pulmonary Medicine Weihai Municipal Hospital Weihai Shandong

**Keywords:** Angiogenesis, apatinib, combination, EGFR‐TKI, NSCLC

## Abstract

**Background:**

The aim of this study was to investigate the feasibility of using a combination of apatinib in the treatment of non‐small cell lung cancer. Apatinib is a tyrosine kinase inhibitor which selectivelyacts on vascular endothelial growth factor receptor 2 (VEGFR‐2) and has shown good efficacy in a variety of malignancies, but the drug resistance is fast in single drug therapy.

**Methods:**

The inhibitory effect of apatinib and other drugs on lung cancer cells was determined by CCK‐8 test in vitro, and the IC50 value was determined. To establish a nude mouse xenograft model, observe the inhibitory effect of apatinib combined with other drugs on lung cancer xenografts in nude mice; immunohistochemical staining of tumor microvessel density and Ki67 expression in transplanted tumor tissues; Western blot analysis of related signaling pathways expression; immunohistochemistry was used to detect tumor microvessel density in other organs and to observe its safety.

**Results:**

In this study, we found apatinib combined with pemetrexed, the first and third generation of epidermal growth factor receptor tyrosine kinase inhibitor, could synergistically inhibit the proliferation of non‐small cell lung cancer cell (NSCLC) lines, reduce the microvessel density and Ki67 protein levels of three non‐small cell lung cancer xenografts, and enhance anti‐tumor activity by synergistically inhibiting the MAPK‐ERK and PI3K‐AKT‐mTOR signaling pathway. Furthermore, there were no pathological abnormalities in the heart, brain, liver and kidney of each group.

**Conclusions:**

The efficacy of apatinib combination is better than that of monotherapy, and there is no significant difference in toxicity of important organs, which suggests the feasibility of a combination of apatinib in the treatment of non‐small cell lung cancer.

## Introduction

Lung cancer is a malignant tumor with the highest mortality, mainly non‐small cell lung cancer.^1^ At present, the treatment of lung cancer has entered the era of targeted therapy. The first targeted drugs applied to the clinic are mainly small molecule tyrosine kinase inhibitors targeting epidermal growth factor and its receptor (EGF‐EGFR) pathway. This pathway promotes tumor proliferation, division and metastasis. However, with the emergence of drug resistance, the therapy for this pathway has entered a bottleneck period, and it is necessary to find new ways to treat tumors. In 1971, Professor Folkman proposed the theory that tumor growth and metastasis depend on neovascularization and that anti‐angiogenesis can be used to treat tumors. Vascular endothelial growth factor (VEGF) is secreted by tumor cells or vascular endothelial cells, and VEGF mediates angiogenesis by binding to vascular endothelial growth factor receptor (VEGFR). Therefore, inhibition of VEGFR signal transduction is an effective therapeutic target in clinical practice. Anti‐angiogenic drugs include anti‐VEGF monoclonal antibodies and VEGFR tyrosine kinase inhibitors (VEGFR‐TKIs).

Bevacizumab is a humanized monoclonal antibody against VEGF, which can block the binding of VEGF to VEGFR and thus reduce tumor angiogenesis.^2^The phase III study of nonsquamous non‐small cell lung cancer in China showed that compared with patients receiving chemotherapy alone, the median progressive survival time of patients receiving first‐line therapy of bevacizumab combined with paclitaxel and carboplatin was 2.7 months longer (9.2 months vs. 6.5 months). The risk of progress was reduced by 60%.^3^ Nintedanib and anlotinib are multiple target tyrosine kinase inhibitors. The results of phase III clinical trials of LUME‐Lung 1 showed that compared with docetaxel combined with placebo, the median progression‐free survival time was significantly prolonged in patients with lung cancer (3.4 vs. 2.7 months, HR = 0.79, *P* = 0.0019).^4^ The results of ALTER0303 test showed that anlotinib could benefit both overall survival (OS) and progression‐free survival (PFS) in the third line treatment of advanced NSCLC.^5^ All these studies confirmed the effect of anti‐angiogenesis.

Apatinib is a selective VEGFR‐2 tyrosine kinase inhibitor. In clinical studies of advanced gastric cancer, apatinib prolonged the OS and PFS of patients with good safety.^6^ It has been approved by the China Food and Drug Administration as a subsequent‐line treatment for advanced gastric or gastric‐esophageal junction adenocarcinoma. In addition, apatinib hepatocellular carcinoma, metastatic knot rectal cancer, thyroid undifferentiated carcinoma and other malignant tumors have shown good results.^7^, ^8^, ^9^ Studies have confirmed that bevacizumab combined with chemotherapy is more effective than a single drug, while the efficacy and side effects of apatinib combined with other drugs are unclear. In this study, three lung cancer cell lines A549, HCC827 and H1975 were selected which represent wild type, EGFR mutant and EGFR mutant with a T790M mutation cell lines. For the type of clinical lung cancer represented by the above three cell lines, the preferred drug is pemetrexed, the first and third passage EGFR‐TKI. We observed the antitumor effects and toxicity of apatinib combined with the above three drugs on three lung cancer cells both in vitro and vivo to investigate the action mechanism of apatinib on NSCLC and provide experimental evidence for the clinical application of apatinib in NSCLC.

## Methods

### Reagents and cell lines

Apatinib mesylate standard products were provided by Jiangsu Heng Rui Pharmaceutical Co. Ltd. (Jiangsu, China) and icotinib standard product was obtained from Zhejiang Beida Pharmaceutical Co., Ltd. (Zhejiang, China), pemetrexed was provided by Qilu Pharmaceutical Co., Ltd. (Shandong, China) and osimertinib standard was purchased from Selleck.cn. Three cell lines A549, HCC827 and H1975 were purchased from the cell bank of the Chinese Academy of Sciences (Shanghai, China) and cultured in RPMI media (HyClone) containing 10% fetal bovine serum (FBS, Gibco),100 U/mL penicillin (HyClone) and 100 ug/mL streptomycin (HyClone). Primary antibodies against AKT(ab8805), phosphor‐AKT(ab8932), ERK(ab54230), phospho‐ERK(ab201015), CD31(ab28364), mTOR(ab2732), phosphor‐mTOR(ab109268) and GAPDH(ab181602) were purchased from abcam (Cambridge, MA, USA). Primary antibodies against ki67 (9449) and anti‐rabbit or anti‐mouse IgG horseradish peroxidase (HRP)‐linked secondary antibodies were purchased from Cell Signaling Technology (Boston, MA, USA).

### Animals

BALB/c‐nu nude mice, 4–5 weeks old, female, weight 18–21 g, were purchased from Nanjing Institute of Biomedicine, Nanjing University and raised under specific pathogen‐free (SPF) conditions. The food and water of nude mice were autoclaved and added once every three days, and the pad was cleaned and replaced once a week.

### Cell viability assays

A549, HCC827 and H1975 cells were treated with different doses of apatinib (2.5, 5, 10, 20, 40, 80 umol/L) or pemetrexed (0.004, 0.02, 0.10, 0.50, 2.50, 12.50 umol/L), icotinib (0.0025, 0.01, 0.04, 0.16, 0.64, 2.56 umol/L), osimertinib (0.0025, 0.001, 0.004, 0.016, 0.064, 0.256 umol/L) for 48 hours. After adding 10 uL CCK8 agent (BestBio, Shanghai, China) to each well and incubating for two hours, the optical density (OD) was then measured at 450 nm. Cell viability was calculated according to the following formula: Cell viability (%) = (OD [experiment] ‐OD [bank])/ (OD [control] ‐OD [bank]) × 100%. The inhibitory concentration (IC_50_) was calculated by using Graph Pad Prism. Combination Index (CI) was calculated by the eq. CI = (CI1/I1) + (CI2/I2), CI < 1 is indicated for synergism.^10^

### Xenograft model

We subcutaneously injected 5 × 10^6^ cells into the right armpit of each nude mouse. The model was successfully prepared after the subcutaneous tumor of nude mice reached 100–150 mm^3^. There was no significant difference in the average volume of transplanted tumors between the nude mice before intervention. A total of 24 tumor‐bearing nude mice from each of the three cell lines were randomly divided into four groups which were control, apatinib, pemetrexed/icotinib/osimertinib and the combined group according to a random number table. Apatinib (Apa), icotinib (Ico) and osimertinib (Osi) were given orally, and pemetrexed (Pem) was given intraperitoneally. The dosage of the combined group was the same as that of the single drug group. The administration time was 21 days (Table 1). The mice bodyweights and tumor volume (width^2^ × length/2) were determined every three days. At harvest the mice were sacrificed under anesthesia, and the heart, brain, liver and kidneys were fixed with 10% formalin. HE staining and immunohistochemical staining were then performed. The transplanted tumor was partially fixed with 10% formalin and then stained with immunohistochemical staining. Some of the tumors were frozen at −80°C for western blot.

**Table 1:** Dosage and method of administration in tumor‐bearing animals

Xenograft model	Compound	Dose (mg/kg)	Route and schedule
A549	Apa	100	p.o. qd*21 days
	Pem	250	i.p. qw*3 wweeks
	Pem + Apa	100 + 250	
HCC827	Apa	100	p.o. qd*21 days
	Ico	60	p.o. qd*21 days
	Ico + Apa	100 + 60	
H1975	Apa	100	p.o. qd*21 days
	Osi	100	p.o. qd*21 days
	Osi + Apa	100 + 100	

i.p., intraperitoneally; p.o, per os (by mouth); qd, once a day; qw, once a week.

### HE and immunohistochemical staining

The fixed transplanted tumor tissue and the heart, brain, liver and kidney tissues were embedded in paraffin and cut into 4 μm serial sections. After the tumor tissue sections were repaired by antigens, CD31 and Ki67 primary and secondary antibodies were incubated and stained according to the manufacturer's instructions and then sealed with neutral gum. The heart, brain, liver and kidney tissue sections were dewaxed and stained with hematoxylin‐eosin (HE) staining kit for HE staining. The pathological changes were observed under light microscope. The CD31 staining method of heart, brain, liver and kidney tissue sections is the same as above. The microvessel density (MVD) was determined by analyzing the expression of CD31. According to the method reported by Weinder,^11^ the count of MVD first selected high blood vessel density regions (hot spots) under a low‐power microscope (×40). The vessel density of three different regions of the hot spot was then counted in a 200‐fold field of vision, and the average value was taken. When the diameter of microvessel was >50 μm, the muscular layer in the wall was not counted. Ki67 positive cells were selected from three regions under a 200‐fold field of view, the percentage of positive cells was counted, and the average value was taken.

### Western blot analysis

Three transplanted tumor tissues were selected for each group which were lysed by radio immunoprecipitation assay (RIPA, Beyotime, China) buffer containing phenyl methane sulfonyl fluoride (PMSF, Solarbio, China) with mild sonication. The concentrations of total proteins were measured by the BCA Protein Assay Kit (Beyotime, China). Equal amount of protein was subjected to 10% sodium dodecyl sulfate polyacrylamide gel electrophoresis (SDS‐PAGE) and blotted on polyvinylidene fluoride (PVDF, Millipore, Billerica, USA). Protein bands were visualized via enhanced chemiluminescence (ECL, Millipore, USA) and analyzed using the Western Blot imaging system (AI600 images, GE, USA), followed by measurement of the density of each band using Image J software.

### Statistical analysis

All data are expressed as the mean ± standard error of the mean (SEM). Data were analyzed using SPSS 20.0 software and mapped by GraphPad Prism 5.0. A two‐tailed unpaired Student's *t*‐test was performed to compare two data sets. Multiple comparisons were tested with two‐way ANOVA followed by Bonferroni's post‐test. A *P*‐value of less than 0.05 was considered statistically significant.

## Results

### The effect of apatinib on three NSCLC cell lines in vitro

**Figure 1:**
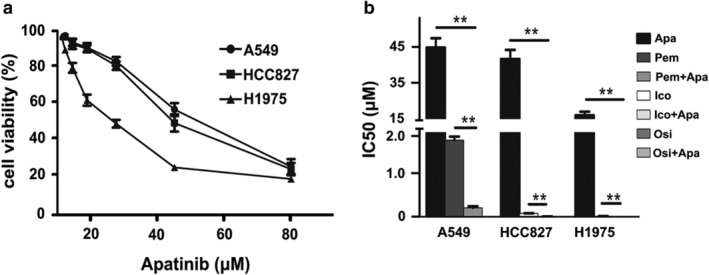
Sensitivity of different drugs to A549, HCC827 and H1975 cells. (**a**) Inhibition of three NSCLC cells by apatinib monotherapy. (**b**) Apatinib combined with pemetrexed, icotinib or osimertinib inhibit proliferation for three NSCLC cells. In the combined group, the concentrations of apatinib for A549, HCC827 and H1975 were as follows: 22, 20 and 8 μmol/L (** *P* < 0.01).

The IC_50_ values of each drug to three NSCLC cell lines are shown in Table 2. The inhibitory effects of apatinib on three NSCLC cell lines were enhanced with increasing drug concentration, and H1975 cells were most sensitive to apatinib (*P* < 0.001, Fig 1(a)). The inhibition of proliferation in the combination group was stronger than that in the single drug group (*P* < 0.01, Fig 1(b)). In A549 cells, the CI of apatinib combined with pemetrexed was 0.61 ± 0.07, in HCC827 cells, the CI of apatinib combined with icotinib was 0.53 ± 0.05, in H1975 cells, the CI of apatinib combined with osimertinib was 0.60 ± 0.05. All have synergistic effects.

**Table 2:** IC_50_ values of each experimental drug to three lung cancer cells (μmol/L, mean ± SD)

	A549	HCC827	H1975
Apa	44.5933 ± 3.9511	39.8800 ± 3.7706	16.4967 ± 1.4775†
Pem	1.9087 ± 0.0900	/	/
Pem + Apa	0.2235 ± 0.0403‡	/	/
Ico	/	0.0839 ± 0.0051	/
Ico + Apa	/	0.0087 ± 0.0024§	/
Osi	/	/	0.0144 ± 0.0014
Osi ± Apa	/	/	0.0017 ± 0.0004¶

† Compared with A549 and HCC827 cell lines, *P* < 0.001.

‡ Compared with pemetrexed single drug group, *P* < 0.001.

§ Compared with icotinib single drug group, *P* < 0.001.

¶ Compared with osimertinib single drug group, *P* < 0.001.

### Antitumor activity of apatinib in vivo

#### Growth of subcutaneous xenografts in nude mice

**Figure 2:**
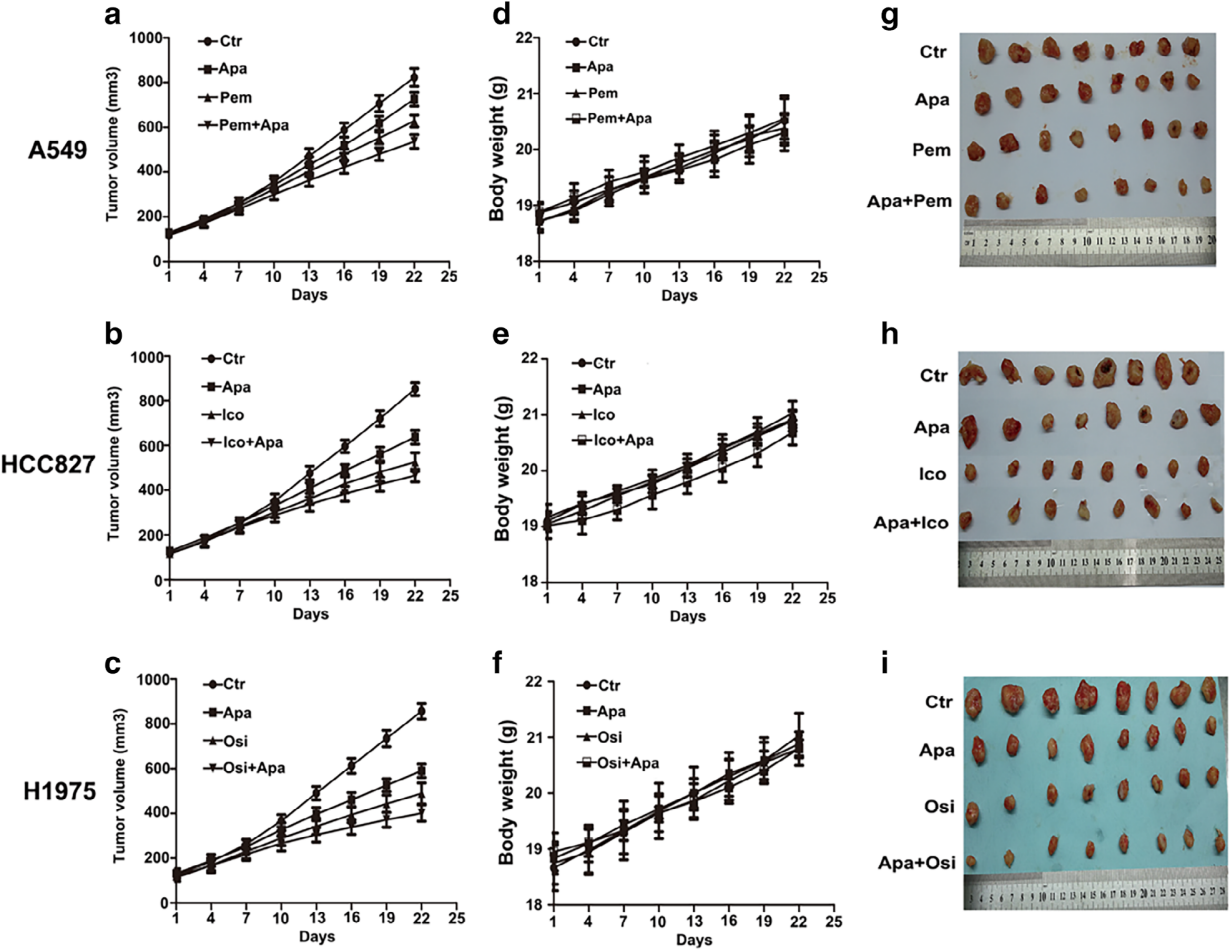
The effect of apatinib combined with pemetrexed, icotinib and osimertinib on three NSCLC xenografts in nude mice. (**a**, **b**, **c**) The growth curves of three xenografts. (**d**, **e**, **f**) Changes in bodyweight of nude mice (**g**, h, i). Photographs of 22 days after treatment of three NSCLC nude mice xenografts.

On the first day of treatment, there were no significant differences in the volume of transplanted tumors among each group of the three human NSCLC nude mice translated tumor models (*P* > 0.05). From the volume growth curve of transplanted tumor in nude mice (Fig 2(a), (b), (c)), compared with the control group, apatinib inhibited the growth rate of transplanted tumors (*P* < 0.05), of which H1975 transplanted tumor inhibition was stronger than A549 and HCC827 xenografts (*P* < 0.05). The growth rate of the transplanted tumors in the combined group was slower than that in the control group and the single‐drug group (*P* < 0.05, Fig 2(a), (b), (c)). Apatinib combined with pemetrexed, icotinib and osimertinib had synergistic inhibitory effects on the growth of three xenografts (CI < 1). In addition, there was no significant weight loss (Fig 2(d), (e), (f)) or treatment‐related death in nude mice after combined therapy.

#### Immunohistochemical staining

**Figure 3:**
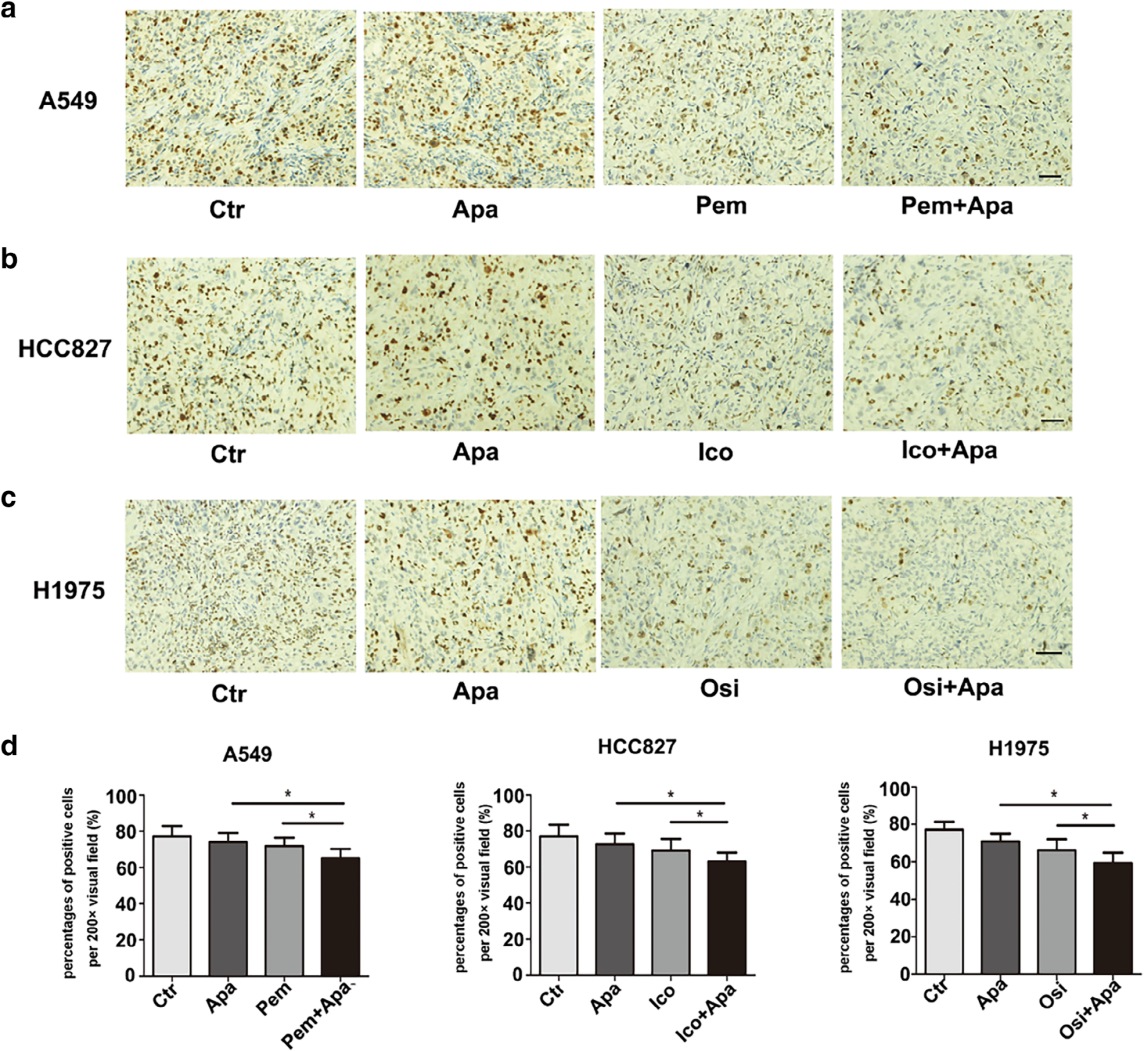
The expression levels Effect of Ki67 in three xenografts. (**a**, **b**, **c**) Representative images of Ki67 positive cells in three xenografts (200×). (**d**) The percentage of ki67 in each field of view. (* *P* < 0.05). Scale bar: 55 μm.

**Figure 4:**
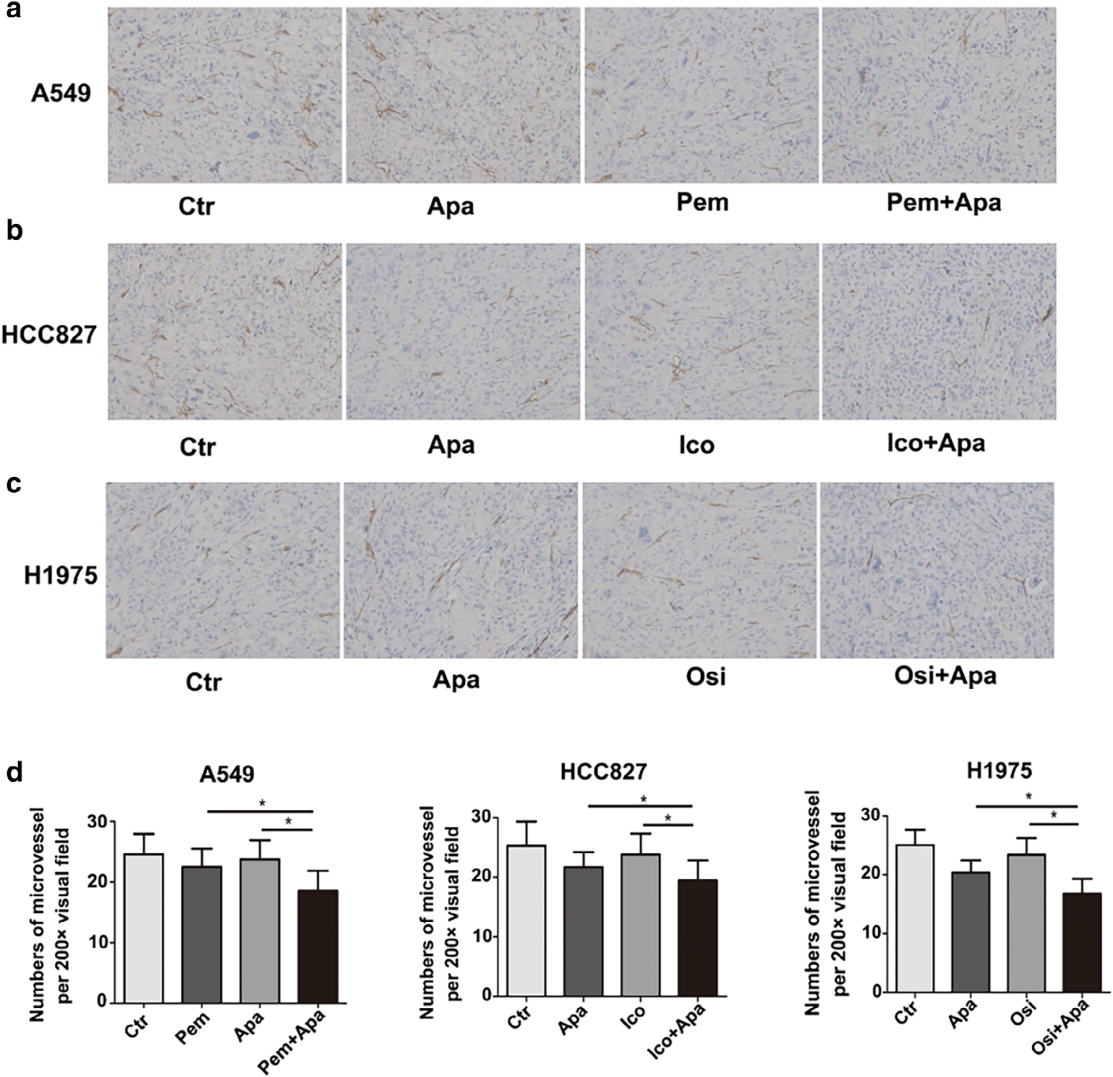
Effects of apatinib, pemetrexed, icotinib, osimertinib alone and its combination on angiogenesis of three xenografts. (**a**, **b**, **c**) Representative images of microvessels stained with CD31 antibody in three NSCLC xenografts (200×). (**d**) The average number of MVD per field of view. (*P < 0.05). Scale bar: 50 μm.

Compared with the control group and the single drug group, the level of Ki 67 in the combined group was significantly lower (Fig 3, *P* < 0.05) and the number of microvessels in the apatinib monotherapy group was significantly decreased (Fig 4, *P* < 0.05); while in the pemetrexed, icotinib and osimertinib monotherapy groups, there were no significant decreases (*P* > 0.05) in the three NSCLC xenografts. In addition, the number of microvessels in the combination group was significantly lower than that in apatinib monotherapy group (*P* < 0.05).

#### Related signal transduction pathways

**Figure 5:**
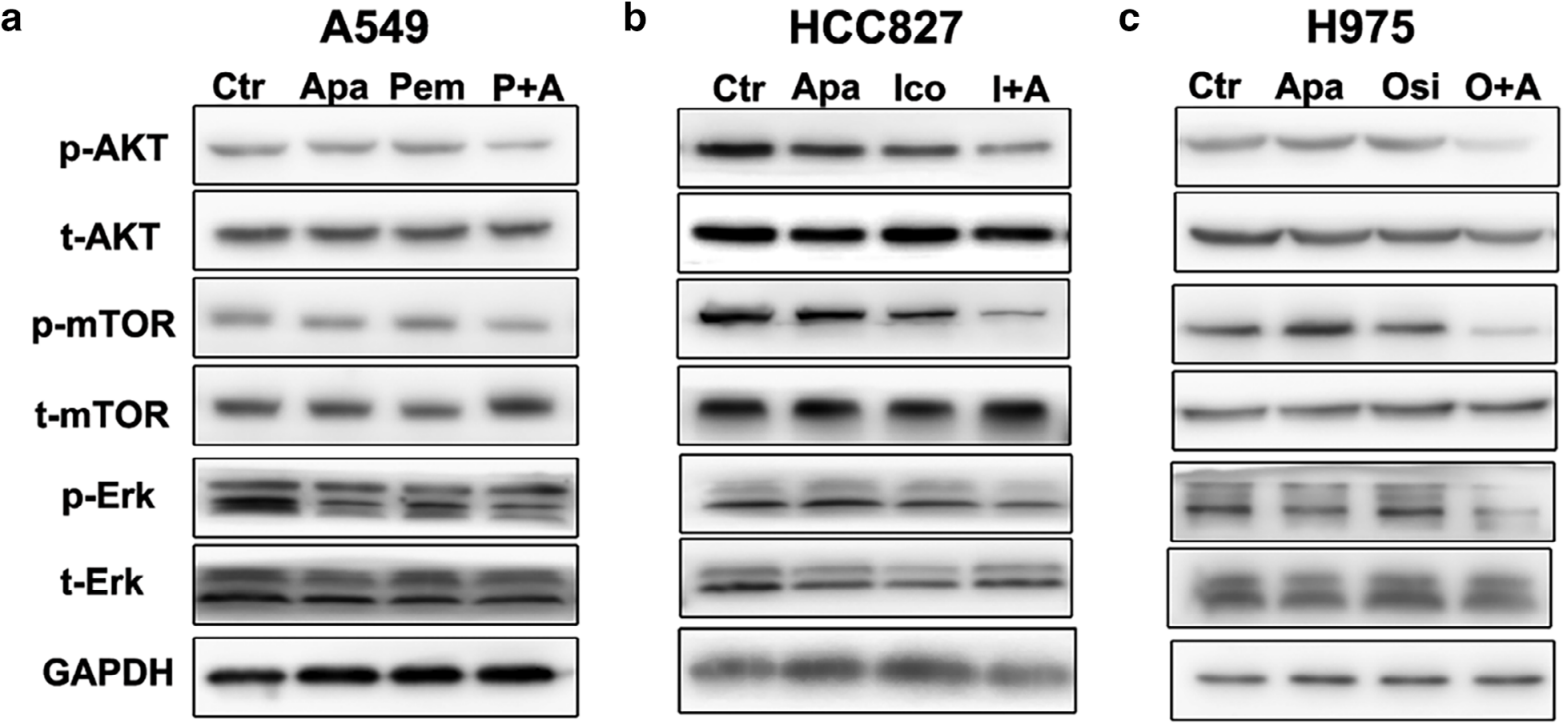
Effects of apatinib combined with pemetrexed, icotinib or osimertinib on EGFR and VEGFR related signal transduction pathways in three xenografts. Blots are representative of three independent experiments.

The expression levels of phosphorylated rapamycin (p‐mTOR), phosphorylated protein kinase B (p‐AKT) and extracellular signal‐regulated kinase (p‐ERK) in HCC827 and H1975 nude mice were significantly lower than those in the control group. However, there was no significant difference between the combined group and the control group in A549 transplanted tumor tissue (Fig 5).

#### Adverse reactions

**Figure 6:**
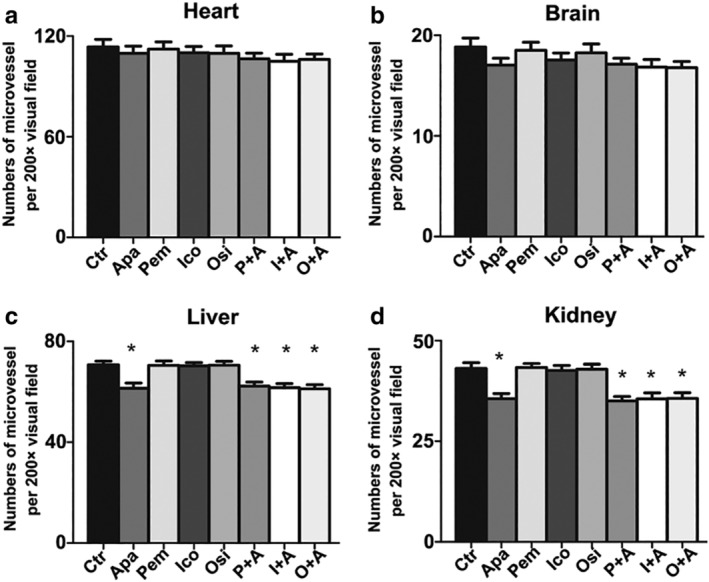
The effect on the microvessel density of heart, brain, liver and kidney in three xenografts. (**a**, **b**, **c**, **d**) are the average number of microvessels for each field of view of heart, brain, liver and kidney tissues, respectively. (200×).

No metastasis was found in the hearts, brains, livers and kidneys of the nude mice, and no abnormal pathological changes were observed by HE staining after treatment. Compared with the control group, there were no significant differences in MVD in the hearts and brains of each drug group (*P* > 0.05). Compared with the control group, pemetrexed monotherapy group, icotinib monotherapy group and osimertinib monotherapy group, MVD in kidney and liver of nude mice was decreased in the apatinib group and the combination group (*P* < 0.05). However, the MVD in the heart, brain, liver and kidney was not significantly decreased in the apatinib combination group compared with that in the apatinib monotherapy group (*P* > 0.05, Fig 6).

## Discussion

The efficiency of antiangiogenic targeting therapy is high; however, drug resistance occurs quickly, and the improvement of OS is limited.^12^ Combination therapy may be more effective and delay the occurrence of drug resistance. Apatinib is a selective VEGFR‐2 inhibitor that can be taken orally. Studies by Tian *et al*. showed that apatinib combined with docetaxel may effectively inhibit the growth of H460 (lung cancer) xenografts in nude mice.^13^ Peng *et al*. found that in the human nasopharyngeal carcinoma xenograft model, the simultaneous application of apatinib and cisplatin had a synergistic effect, while sequential administration showed only an additive effect.^14^ Studies by Feng *et al*. confirmed that in the A549 nude mice transplantation model, apatinib enhanced the antitumor effect of docetaxel.^15^ Li *et al*. found that in vitro, apatinib combined with gefitinib enhanced the inhibitory effect on four NSCLC cell lines (A549, HCC827, H1975 and H1650). In the H1975 nude mice model, apatinib combined with gefitinib could inhibit tumor growth, inhibited the activation of EGFR and VEGFR2, and decreased the level of CD31 and vascular endothelial growth factor A. In patients with gefitinib resistance, apatinib combined with gefitinib achieved a median PFS of 4.60 months.^16^ These findings suggest that apatinib is expected to be an anti‐tumor drug for NSCLC, and combination chemotherapy or first‐generation epidermal growth factor receptor tyrosine kinase inhibitor (EGFR‐TKI) may be more effective. Apatinib combined with the third generation EGFR‐TKI‐osimertinib has not previously been reported, and the side effects of combination therapy are unknown.

**Figure 7:**
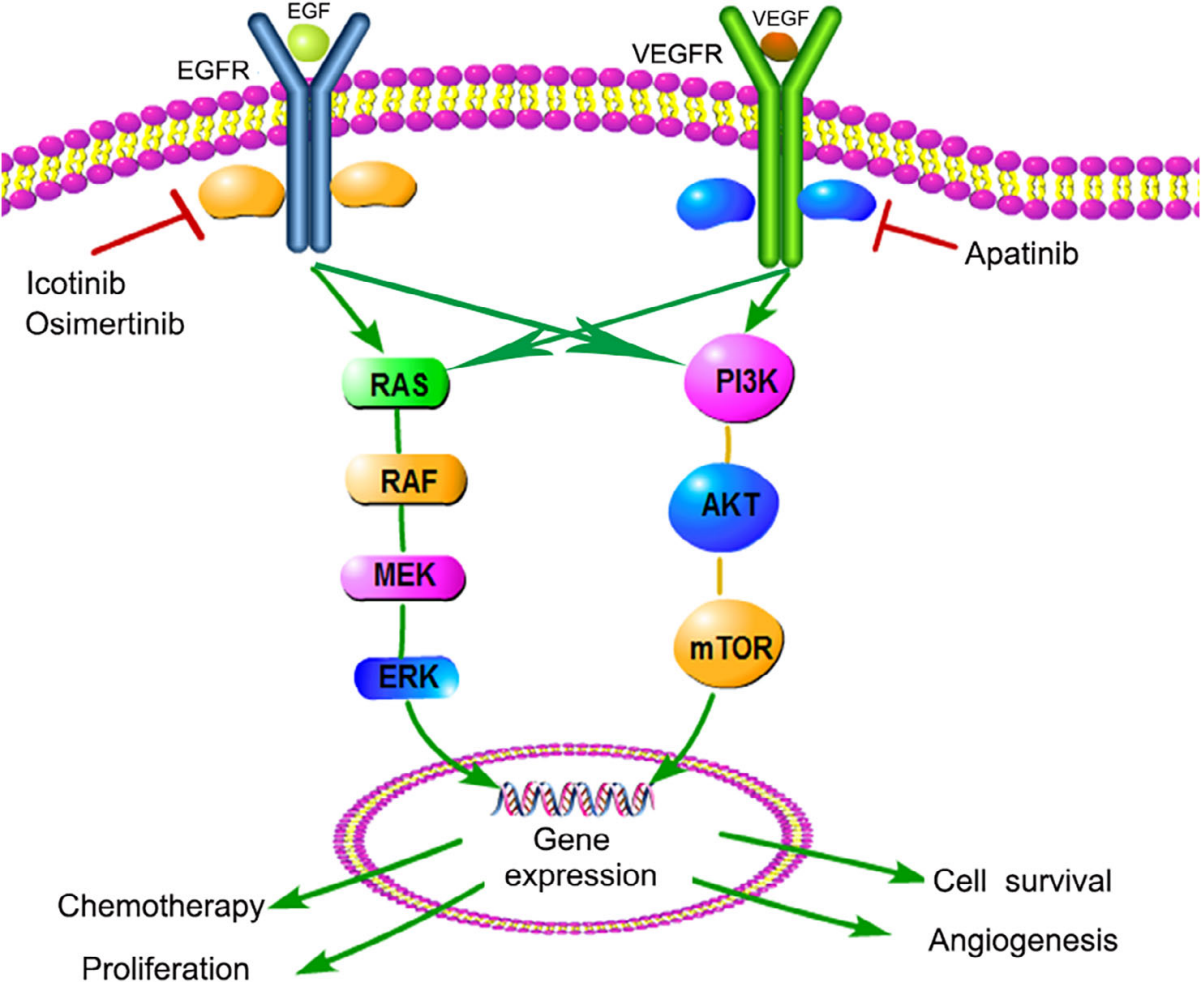
Effect of VEGFR and EGFR on downstream signaling pathways.

Our results showed that in vitro, H1975 cell line was more sensitive to apatinib than A549 and HCC827 cell line. Apatinib combined with pemetrexed can synergistically inhibit the proliferation of A549 cell line, and apatinib combined with icotinib can enhance the inhibition of HCC827 cell line, which is consistent with other studies. In addition, for the first time, our study found that the inhibitory effect of apatinib combined with osimertinib in the proliferation of H1975 cell line was significantly better than that of the osimertinib single drug, which may provide a solution for overcoming the drug resistance of osimertinib. Apatinib (100 mg/kg/day) alone was found to significantly inhibit the growth and angiogenesis of transplanted tumors in three NSCLC nude mice models, and the inhibitory effect of apatinib combined with EGFR‐TKI in H1975 xenograft was apparent. The inhibitory effect of apatinib combined with chemotherapy or EGFR‐TKI was better than that of single drug treatment, which was consistent with our results in vitro. Activation of the PI3K/AKT/mTOR and Ras/Raf/MEK/ERK signaling pathways have been shown to play a key role in cell survival at all stages of cancer.^17^ Studies have reported that the most important signaling pathways are AKT and ERK.^18^Apatinib can effectively reduce the phosphorylation level of key proteins in the above signaling pathways, thereby further reducing the phosphorylation of eukaryotic initiation factor 4E1, which plays an important role in promoting tumor cell proliferation and inhibiting apoptosis and neovascularization.^19^ Our results indicate that apatinib combined with EGFR‐TKI has better multiple inhibition of cell proliferation‐related signaling pathways than single agents. Apatinib can significantly enhance the anti‐tumor effect of chemotherapeutic drugs or EGFR‐TKI on NSCLC. The molecular mechanism of the synergistic effect remains to be elucidated. We speculate that: (i). Chemotherapeutic drugs can induce apoptosis, leading to a decrease in VEGF secretion and a decrease in VEGFR‐2 expression, thereby inhibiting angiogenesis and further tumor growth, and (ii) EGFR and VEGFR2 may synergistically inhibit tumors by inhibiting the MAPK‐ERK and PI3K‐AKT‐mTOR pathways (Fig 7). Tonra *et al*. showed that EGFR and VEGFR2 have a synergistic anti‐tumor effect in a preclinical model of pancreatic cancer and colon cancer. Its possible mechanism is to reduce the expression of VEGF through the mechanism of both hypoxia inducible factor (HIF)‐1‐independence and HIF‐1 dependence.^20^ Anti‐angiogenic therapy not only reduces the formation of new blood vessels, but also induces tumor vascular normalisation,^21^ thereby increasing the local drug concentration of the tumor and enhancing the anti‐tumor activity. A series of preclinical and clinical evidence support the inhibition of VEGF signaling pathways that transiently normalize tumor blood vessels.^22^, ^23^, ^24^, ^25^, ^26^ The development of multidrug resistance is the main reason for the failure of cancer treatment. The drug efflux mediated by transmembrane protein ATP‐binding cassette (ABC) transporter family is related to drug resistance to common chemotherapeutic agents.^27^ Studies have shown that apatinib can reverse multidrug resistance by inhibiting the efflux function of multiple ABC transporters, such as multidrug resistance protein 1 (ABCB1) and breast cancer resistance protein (ABCG2).^28^

Tolerance and side effects are the primary considerations for combination therapy. Kamba *et al*. have found that a certain dose of VEGF inhibitor can reduce the microvessels of thyroid, pancreas, kidney and other organs of normal mice, but will not cause damage to their health, only corresponding changes in physiological function. In addition, the sensitivity of normal mice to VEGF inhibitors was much lower than that of tumor‐bearing mice, and the blood vessels were able to regenerate after withdrawal of the drug.^29^ Our study indicated that no obvious pathological damage was found in the vital organs of the heart, brain, liver or kidney of nude mice after treatment with apatinib combined with chemotherapy or EGFR‐TKI, and the MVD of the heart and brain did not change significantly; however, the MVD of the liver and kidney was significantly decreased. Compared with the apatinib monotherapy group, there was no significant decrease in the MVD of heart, brain, liver and kidney in the apatinib combined with chemotherapy or EGFR‐TKI group, indicating the feasibility of combination therapy.

In conclusion, apatinib combined with chemotherapeutic agent or EGFR‐TKI can improve anti‐tumor activity, which may have the potential of delaying drug resistance and prolonging the survival time of NSCLC patients. Some clinical studies have confirmed that some small molecule anti‐angiogenic drugs combined with other treatments can improve the overall survival of patients with lung cancer.^30^ Apatinib alone could significantly inhibit the growth of H1975 cell line and xenograft in nude mice, which indicates that apatinib had a better effect on NSCLC containing L858R and T790M double mutant, and may have some enlightening effect on clinical application of apatinib. The therapeutic effect of apatinib should be further evaluated in clinical practice.

## Disclosure

None declared.
